# Epidemiology of Hip Fractures in Two Regions of Ukraine

**DOI:** 10.1155/2018/7182873

**Published:** 2018-06-03

**Authors:** V. V. Povoroznyuk, N. V. Grygorieva, J. A. Kanis, E. V. McCloskey, H. Johansson, S. S. Strafun, M. O. Korzh, V. M. Vaida, F. V. Klymovytsky, V. S. Forosenko, R. O. Vlasenko

**Affiliations:** ^1^State Institution «D. F. Chebotarev Institute of Gerontology NAMS Ukraine», Ukrainian Scientific-Medical Center of Osteoporosis, Kyiv 04114, Ukraine; ^2^Centre for Metabolic Bone Diseases, University of Sheffield Medical School, Beech Hill Road, Sheffield S10 2RX, UK

## Abstract

Worldwide, the number of hip fractures, the most important osteoporotic complication in the elderly, continues to increase in line with the ageing of the population. In some countries, however, including the Ukraine, data on the incidence of hip fracture are limited. This article describes the first analysis to characterize the incidence of hip fracture in the Ukrainian population from the age of 40 years. It is based on data from two regional studies, namely, the Vinnitsa city study and the STOP study, which were performed during 1997–2002 and 2011-2012 years, respectively. Hip fracture incidence rates were demonstrated to increase with increasing age. The rates were higher among younger men than women, however, with a female preponderance from the age of 65 years upwards. The incidence of hip fractures in Ukraine is 255.5 per 100,000 for women aged 50 years and older and 197.8 per 100,000 for men of the corresponding age. Overall, the incidence of hip fracture was comparable with data from neighboring countries, such as Poland and Romania. Hip fractures constitute a serious healthcare problem in Ukraine, and changes in healthcare are required to improve the management and long-term care of osteoporosis and its complications.

## 1. Introduction

Osteoporosis, a systemic skeletal disease characterized by low bone mass and microarchitectural deterioration of bone tissue with a resulting increase in bone fragility and low trauma fractures, [1] is a considerable public health issue. Osteoporotic fractures occur in one in three women and one in five men over the age of fifty in Western Europe. Fractures of the hip, vertebral body, proximal humerus, and distal forearm are commonly termed “major osteoporosis fractures,” with hip fracture in particular associated with a high impact on morbidity, mortality, and health expenditure worldwide [2–4].

The incidence of hip fracture progressively increases with age, approximately doubling for each subsequent decade after age 50, and is 2-3 times higher in women than in men [5–7]. Indeed, 90% of all hip fractures occur in people older than 50 years [8], and as older age groups are the most rapidly expanding in the population, the number of hip fractures can also be expected to increase, even if the age-related incidence of hip fractures remains unchanged. The number of hip fractures worldwide is expected to increase from 1.7 million in 1990 to 6.3 million in 2050. Assuming that the age-related incidence might increase by only 1% per year, the number of hip fractures around the world would reach 8.2 million in 2050 [8].

The incidence of hip fracture, as well as other osteoporotic fractures, varies markedly around the world [9–11] and underlies the need for differently calibrated FRAX tools for individual countries. As well as allowing the development of country-specific FRAX models, better characterization of the epidemiology of hip fractures within countries can, more importantly, inform and influence healthcare planning. A previous study in the Ukraine suggested that bone mineral density (BMD) differed across regions [12], but no studies have previously documented the epidemiology of hip fractures. The aim of the present study was therefore to estimate age- and sex-specific hip fracture rates in Ukraine.

## 2. Materials and Methods

Ukraine, in Eastern Europe, shares borders with the Russian Federation to the east and northeast; Belarus in the northwest; Hungary, Slovakia, and Poland in the west; and, finally, Romania and Moldova in the southwest. It comprises 24 provinces and the present study was performed within two of these.

### 2.1. Study Design and Participants

The first study was retrospective one which had been approved by “Ethics Committee of D. F. Chebotarev Institute of Gerontology NAMS” (2005/02/15) and performed at 2005-2006 in the city of Vinnitsa. Hip fracture data was gathered in the years 1997–2002 inclusively. Vinnitsa is located in west-central Ukraine and is the administrative center of the Vinnitsa region.

The second study (study of the prevalence of osteoporotic fractures in Ukrainian population, STOP study) was organized by the Ukrainian Association of Osteoporosis, with the support of the Ukrainian Association of Orthopaedics, approved by “Ethics Committee of D. F. Chebotarev Institute of Gerontology NAMS” (2013/06/11), and collected hip fracture data for two years from the beginning of 2011 to the end of 2012. The study had two centers, one in the Vinnitsa area, but excluding Vinnitsa city, and one in the city of Uzhhorod, the administrative center of the Zakarpattia region located in Western Ukraine near the Slovakia border.

In both studies, cases were defined as those patients (aged 40 years or more) who were hospitalized for a hip fracture (International Classification of Diseases, Tenth Revision (ICD10) code S72.0 [femoral neck], S72.1 [trochanter], S72.2 [subtrochanteric fracture]) or who refused hospitalization and surgical treatment and applied only to the ambulance departments (a few patients). Patients with a code of S72.9 (unspecified site of femoral fracture) were excluded unless the accompanying surgical procedure indicated surgery on the hip. All hip fracture cases were included, regardless of the country of origin of the patient or the degree of trauma, but cases associated with neoplasia were excluded.

Data were gathered from multiple sources (ambulance registers, city and district hospitals registers, and outpatient/clinic registers), and multiple admissions for the same fracture were deleted before analysis to avoid double or multiple counting. The present analysis was confined to hip fractures occurring from the age of 40 years upwards, since this is the lowest age limit used in FRAX.

### 2.2. Data Access and Cleaning Methods

The authors had direct access to all databases.

Duplicated data (recurrent admissions of the same patient or continued treatment in the other facility) were identified and removed from analysis.

### 2.3. Statistical Methods

Incidence rates were estimated as the number of men and women in 10-year age intervals with at least one hip fracture in the study year divided by the age- and sex-specific population of the region. Since the age interval of 40–49 years was not available for Vinnitsa city (1997–2002) and no fractures occurred in that interval in Uzhhorod city, log-linear regression was used to extrapolate the incidences below the age of 50 years. To compute the national incidence of hip fractures, incidence rates derived using the local populations were then merged by weight and extrapolated to the population of Ukraine using government estimates for the same year.

## 3. Results

### 3.1. Vinnitsa City Study

**Figure 1:**
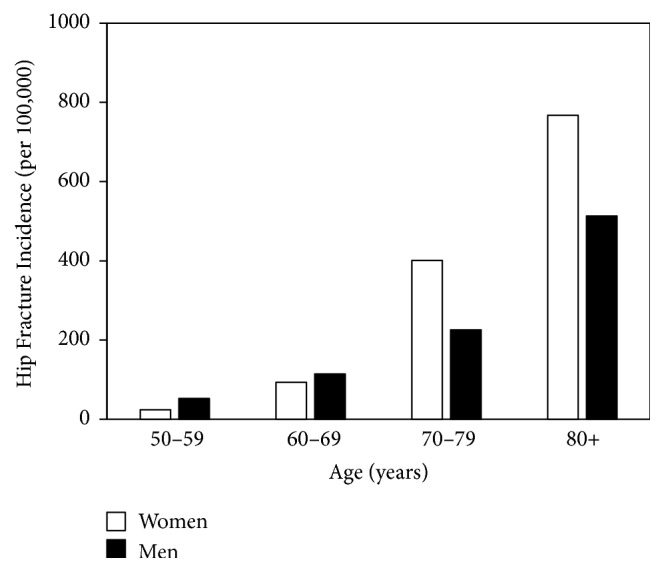
Hip fracture incidence per 100,000 in men and women in Vinnitsa city, across the age bands shown, for data collected across 1997–2002 inclusively.

The numbers of incident hip fractures and the size of the relevant population at risk, in 10-year age bands, in the Vinnitsa city study are shown in Table 1. The number of women in the population was greater than the number of men within each age band, a difference that was particularly marked in the oldest age group (80+ years). The incidence of hip fracture rose with age for both men and women (Figure 1). Within the whole 6 years of the study, the incidence of hip fractures was 56% higher in women than men, but there were important interactions with age; thus, hip fracture incidence was higher at younger ages in men (2.2-fold at ages 50–59 years and 1.2-fold at 60–69 years), while the rates were higher in women at older ages (1.8-fold at ages 70–79 years and 1.5-fold at ages 80+ years) (Figure 1).

**Figure 2:**
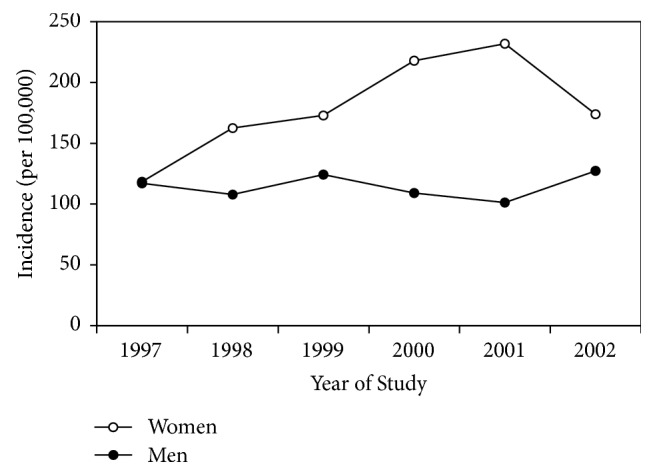
Hip fracture incidence in men and women aged 50 years and older in Vinnitsa city over the years 1997–2002 inclusively.

When data were adjusted for age, there was no change in incidence with calendar year for men (Hazard Ratio (HR) per year 0.99, 95% CI: 0.92–1.07, *p* > 0.30) (Figure 2). In contrast, rates in women showed a statistically significant increase with later calendar years (HR per year 1.07, 95% CI: 1.02–1.13, *p* = 0.0073) (Figure 2).

### 3.2. STOP Study

The number of hip fractures and calculated incidences within the two centers of the STOP study during the observation period of 2011-2012 are shown in Table 2. As expected, the overall incidence rates in those aged 40 and older were higher in women than in men and increased with age in both genders. As observed in the Vinnitsa city study above, the incidence of hip fractures was higher in men than in women at younger ages, but this pattern was reversed at older ages. This inversion occurred at an earlier age in the Vinnitsa area (50–59 years) than in Uzhhorod city (70–79 years) (Table 2). While the hip fracture rates in men and women aged 50 years and older were similar across both sites, the rates at the oldest age (80+ years) appeared substantially higher in Uzhhorod than in the Vinnitsa area.

**Figure 3:**
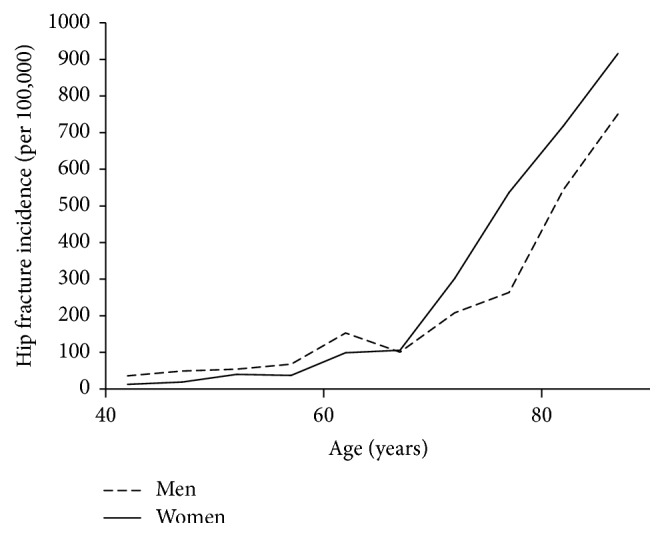
Hip fracture incidence in men and women aged 40 years and older in the Ukraine. Data are computed in 5-year age intervals.

The incidence rates for hip fracture within the Ukraine, computed by combining all three data sets and adjusting to the age distribution of Ukraine, are shown in Figure 3. The incidence was higher in young men but was higher in women from the age of 67 years upwards. For example, at 50 years of age, men had a 35% higher incidence of hip fracture, while at 80 years of age, the rate in men was 24% lower than that in women. The impact of gender was substantially smaller than that of age. Thus, in women, the incidence rate at 80 years of age was 18-fold higher than that at 50 years of age; in men the ratio was 10-fold between the same ages (Figure 3).

From UN population statistics, we estimate that the number of hip fractures in Ukraine in 2020 will be approximately 28.74 (73% in women), having risen from a total of 22 200 in 2000 and projected to reach 36 120 in 2050, a 63% increase from 2000.

## 4. Discussion

This study characterizes for the first time the hip fracture incidence in the Ukraine from the age of 40 years upwards, based on data from two regional studies performed in 1997–2002 and 2011-2012. As expected, hip fracture incidence increased progressively with age in both sexes. At younger ages, incidence rates were higher in men than in women but were substantially higher in women at older ages. The majority of hip fractures occurred in men and women aged 70 years or more. Overall, the incidence of hip fractures in Ukraine is 255.5 per 100,000 for women aged 50 years and older and 197.8 per 100,000 for men of the corresponding age.

Ukraine, one of the biggest countries in Europe, has more than 44 million citizens with approximately 1 in 6 (15.6%) currently aged 65 years and above (almost 7 million people). Demographic projections indicate that this subgroup of the Ukrainian population will increase to 19.9% in 2030 and 23.3% in 2050. Likewise, the population aged 80 years or more will also grow; estimates suggest that the female population aged 80 and older will increase from 4.9% in 2016 to 7.6% in 2050, with the respective figures for men being 2.1% to 3.0% [13]. The burden of hip fractures will increase in line with the ageing of the population, though estimates may be conservative given the assumption that the age- and sex-specific risk of hip fracture will remain unchanged over this period.

**Figure 4:**
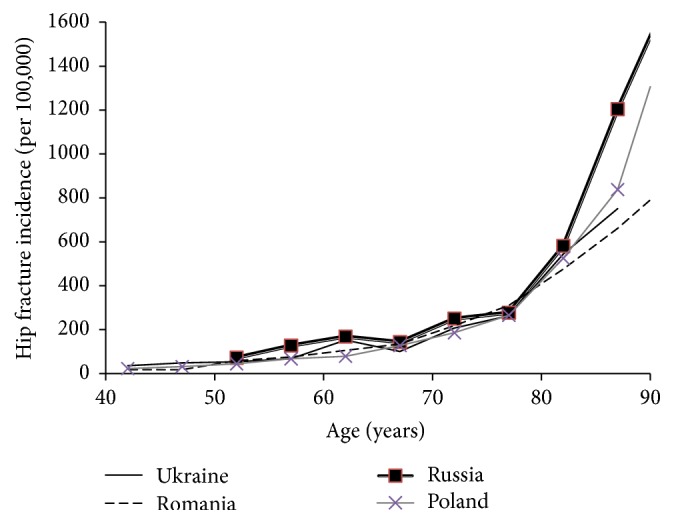
Hip fracture incidence by age in women within Ukraine and in the neighboring countries of Russia, Poland, and Romania.

The hip fracture incidence in women in the Ukraine is very similar to that observed in the neighboring countries of Romania, Poland, and Russia [14–16] (Figure 4). A similar pattern was seen for hip fracture incidence in men (not shown). Based on the age-standardized annual incidence of hip fracture for men and women, Ukraine belongs to the moderate-risk countries for fracture risk [9].

The present study has some strengths and limitations. A limitation is that we cannot be sure that the regions studied, just 2 of over 20 in the Ukraine, are fully representative of the national population. However, the data on hip fracture rates are based on two well-conducted surveys over a reasonable timeframe. Importantly, there appears to be reasonable consistency in the estimates between the regions, and the patterns in men and women are also consistent. Variances in estimates at older age groups may reflect the sample sizes due to the relatively low life expectancy in Ukraine compared to many other European countries. In the absence of additional data, it seems reasonable to work on the assumption of representativeness, particularly as the gender and age distributions are similar to national data (data not shown) and the quality of the data is likely to be good. Importantly, the study design was able to minimize the overidentification of cases (double counting), though it was not possible to exclude pathological fractures or assess the accuracy of reporting or coding of fractures.

## 5. Conclusion

In conclusion, this study for the first time characterizes hip fracture incidence in the Ukrainian population from the age of 40 years. These data have already been utilized to construct a FRAX model for Ukraine as a basis for fracture risk assessment [17]. Furthermore, the study permits the estimation of hip fracture burden in the Ukraine to inform government and healthcare policy. Further studies in other regions of Ukraine will strengthen this knowledge base.

## Figures and Tables

**Table 1:** Total population and the number of hip fractures by age and sex for 1997–2002 in the Vinnitsa city.

Years	1997			1998			1999			2000			2001			2002			Total		
Age (y)	Pop.	Hip Frac.	Incid	Pop.	Hip Frac.	Incid.	Pop.	Hip Frac.	Incid.	Pop.	Hip Frac.	Incid.	Pop.	Hip Frac.	Incid.	Pop.	Hip Frac.	Incid.	Pop.	Hip Frac.	Incid.
Women																					
50–59	19947	4	20.1	20005	5	25	20313	3	14.8	20832	5	24	20456	5	24.4	21771	8	36.7	123324	30	24.3
60–69	16678	11	66	16468	12	72.9	15527	9	58	14710	22	149.6	14356	15	104.5	16275	19	116.7	94014	88	93.6
70–79	8830	32	362.4	8982	35	389.7	9388	39	415.4	9660	48	496.9	10268	52	506.4	11254	28	248.8	58382	234	400.8
80+	3592	11	306.2	3783	28	740.2	3921	34	867.1	4361	33	756.7	4521	43	951.1	4193	38	906.3	24371	187	767.3
Total	49047	58	118.3	49238	80	162.5	49149	85	172.9	49563	108	217.9	49601	115	231.9	53493	93	173.9	300091	539	179.6

Men																					
50–59	18636	14	75.1	18923	9	47.6	19154	8	41.8	19637	12	61.1	19229	4	20.8	19965	14	70.1	115544	61	52.8
60–69	11208	14	124.9	11165	7	62.7	10806	15	138.8	10590	9	85	10544	11	104.3	9648	17	176.2	63961	73	114.1
70–79	4644	8	172.3	4622	10	216.4	4682	12	256.3	4605	14	304	4912	14	285	5741	8	139.3	29206	66	226
80+	1372	6	437.3	1465	13	887.4	1593	10	627.7	1857	5	269.3	1883	8	424.9	1572	8	508.9	9742	50	513.2
Total	35860	42	117.1	36175	39	107.8	36235	45	124.2	36689	40	109	36568	37	101.2	36926	47	127.3	218453	250	114.4

*Note*. Pop.: population size; Hip Frac.: number of incident hip fractures recorded; Incid.: incidence of hip fracture per 100,000 person years.

**Table 2:** Observation time and the number and incidence of hip fractures in women and men in the two centers of the STOP study within the age bands shown. The data for 2011-2012 are combined.

Region	Vinnitsa area						Uzhhorod city					
Sex	Women			Men			Women			Men		
Age (y)	Person years	Hip Frac.	Incid.	Person years	Hip Frac.	Incid.	Person years	Hip Frac.	Incid.	Person years	Hip Frac.	Incid.
40–49	10974	7	63.8	11136	12	107.8	16688	0	0	14315	0	0
50–59	10945	19	173.6	9855	13	131.9	18406	10	54.3	14020	10	71.3
60–69	8482	13	153.3	5867	8	136.4	12673	17	134.1	8718	20	229.4
70–79	8901	29	325.8	4737	14	295.5	7890	35	443.6	4331	8	184.7
80+	5329	19	356.5	1686	8	474.5	3123	53	1697.1	1241	19	1531
Total	44631	87	194.9	33281	55	165.3	58780	115	195.6	42625	57	133.7
50+	33657	80	237.7	22145	47	194.2	42092	115	273.2	28310	57	201.3

*Note*. Hip Frac.: number of incident hip fractures recorded; Incid.: incidence of hip fracture per 100,000 person years.

## Data Availability

All data is stored as electronic databases in Ukrainian Scientific-Medical Center of Osteoporosis, (Kyiv, Ukraine) and can be accessed after a preliminary request to the head of the center.
